# The association between workplace ostracism and knowledge-sharing behaviors among Chinese university teachers: The chain mediating model of job burnout and job satisfaction

**DOI:** 10.3389/fpsyg.2023.1030043

**Published:** 2023-02-01

**Authors:** Guang-Hui Wang, Jia-Hui Li, Hui Liu, Cristina Zaggia

**Affiliations:** ^1^School of Education (Teachers College), Guangzhou University, Guangzhou, China; ^2^Department of FISPPA, University of Padova, Padova, Italy

**Keywords:** workplace ostracism, job burnout, job satisfaction, knowledge-sharing behaviors, conservation of resources theory

## Abstract

Workplace ostracism is an essential predictor of knowledge-sharing behaviors, but few studies have explored the influence of this mechanism in higher education. According to the conservation of resources theory, this study elucidated the roles of job burnout and job satisfaction as sequential mediators of the link between workplace ostracism and knowledge-sharing behaviors in a sample of 388 university teachers. The results of the study were analyzed *via* structural equation modeling (SEM). Higher knowledge-sharing behaviors were associated with lower workplace ostracism, lower job burnout, and more job satisfaction. Furthermore, increased workplace ostracism was associated with more job burnout, but job satisfaction was not related to workplace ostracism. The relationship between workplace ostracism and knowledge-sharing behaviors was mediated by job burnout and was sequentially mediated by job burnout and job satisfaction. These findings help to clarify the mechanisms underlying the association between workplace ostracism and knowledge-sharing behaviors in university teachers. The theoretical and practical implications of the findings are discussed.

## Introduction

In the current knowledge economy, knowledge-sharing behaviors (KSBs) play an increasingly apparent role in higher education, in particular (Tan, 2016; Jalili and Ghaleh, 2021). Universities, as knowledge-intensive organizations, are dominant actors in knowledge creation, knowledge dissemination, and knowledge learning (Al-Kurdi et al., 2020). In addition, as the principal player in knowledge creation and dissemination for university teachers, improving their KSBs can enhance the creativity and core competitiveness of the university and promote appropriate resource allocation (Bibi and Ali, 2017; Javaid et al., 2020). As a core element of the knowledge management process (Alavi and Leidner, 2001), KSBs are extra role behaviors that members of an organization use to share advice, opinions, ideas, and information with each other, mainly by sharing their knowledge, experience, and skills (De Clercq and Pereira, 2020; Nguyen, 2021). KSBs aim to convert existing knowledge and ideas into new knowledge by breaking down barriers and obstacles between different knowledge holders and spreading knowledge from the individual to the organizational level (Shah et al., 2009). However, in practice, most people view knowledge as private property and valuable resources and are more likely to hoard knowledge than share it, especially in stressful situations where individuals are less likely to engage in KSBs (Zhang et al., 2020). It is a dilemma for KSBs that university teachers in higher education institutions are often unwilling to share or deliberately hide knowledge from their colleagues, which results in a massive waste of human resources, increases the cost of acquiring knowledge in institutions, and hinders institutional innovation and change in the long run (Hernaus et al., 2019; Karim, 2020). Meanwhile, although some researchers pointed out several barriers to KSBs in higher education (Al-kurdi et al., 2014; Feiz et al., 2019), there is a lack of empirical research into KSBs in this sector (Al-Kurdi et al., 2020). Therefore, we should pay attention to the antecedents of restraining knowledge sharing of teachers.

In light of this, previous research found that KSBs only work well if people are willing to cooperate (Zaman et al., 2021). Workplace ostracism, a form of “cold violence”, has attracted the attention of scholars as a phenomenon that not only brings about painful emotional experiences and the deterioration of interpersonal relationships but also hinders cooperation and communication among employees of the organization and has a negative impact on KSBs (Zhao et al., 2020; Zaman et al., 2021). The conservation of resources (COR) theory provides a plausible framework for explaining how workplace ostracism affects KSBs from the perspective of individual resource gains and losses. The COR theory proposes that individuals constantly strive to acquire, conserve, and protect resources, regardless of whether they are facing or suffering actual losses of existing resources or failing to receive new resources, which will thus stimulate a stress response. Conversely, when individuals are not under stress, they attempt to obtain new resources to build a strategic reserve; when they are under pressure, they attempt to protect their resources to minimize the loss of resources (Hobfoll, 1989). According to the COR theory, when workplace ostracism is high, ostracized employees will be exhausted and reluctant to share their knowledge in an organizational context (Zhao and Xia, 2017). Thus, we expect university teachers who suffer from workplace ostracism to perceive enormous psychological pressure to engage in less KSB to prevent the further depletion of their resources.

In addition, workplace ostracism can significantly predict job burnout (Qian et al., 2019), which inhibits individuals from engaging in KSBs (Zhang et al., 2016; Ali et al., 2021). It has also been shown that job satisfaction mediates between workplace ostracism and innovative behaviors and that workplace ostracism reduces job satisfaction, thus inhibiting innovative behaviors (Chung and Kim, 2017). Moreover, individuals with high job satisfaction have a greater willingness to participate in prosocial behaviors, including KSBs (Teh and Sun, 2012; Umar et al., 2021). More importantly, a meta-analysis by Madigan and Kim (2021) examined the strength of the relationship between job burnout and job satisfaction. According to the results of the abovementioned study, job satisfaction did not predict job burnout, university teachers with low job satisfaction did not necessarily develop job burnout, and university teachers with high job satisfaction could have burnout, but reducing burnout could simultaneously increase job satisfaction. In addition, a meta-analysis by Lee et al. (2011) revealed that workplace stress is the primary antecedent of job burnout, job satisfaction is a critical outcome of job burnout, and all three dimensions of job burnout are predictive of job satisfaction. Through the literature review, we found out that the research results of workplace ostracism are mostly based on foreign organizational situations. Only a few studies have explored the applicability of workplace ostracism in the organizational context of higher education in China. In addition, there is still a lack of research on the specific mechanisms of workplace ostracism. The secret of how workplace ostracism affects the knowledge-sharing behavior of university teachers has not been opened. As such, our study employs the COR theory as a lens to examine how workplace ostracism affects the KSBs of university teachers and uses job burnout and job satisfaction as mediating variables to investigate the mechanisms underlying the effect of workplace ostracism on KSBs of university teachers. We further examined the chain mediating effects of job burnout and job satisfaction, thereby helping us gain a deeper and more comprehensive understanding of how, in combination, these variables affect the KSBs of university teachers.

## Literature review and hypotheses

### Relationship between workplace ostracism and KSBs

Workplace ostracism is defined as the extent to which an individual perceives that he or she is ignored or excluded by others in the workplace, and the intensity of workplace ostracism perceived by employees is closely related to their subjective feelings (Ferris et al., 2008). Ostracism occurs across different age groups, cultures, and demographic lines and, most significantly, in organizational settings, where ostracizing actions appear in all dimensions (Balliet and Ferris, 2013). Universities are based on social relationships, and they comprise a complex network of interactions where members collaborate to improve their work process and efficiency (Clement et al., 2020). In the context of Eastern cultures, the social networks constructed by prevailing collectivism and interpersonal relationships are unique to universities (Bilal et al., 2020). While the concept of workplace ostracism originated in the organizational behavior field, recent research has also begun to focus on the impact of workplace ostracism in knowledge-based organizations such as universities (Fatima et al., 2019, 2021; Bilal et al., 2021; Karim et al., 2021). Unlike many negative workplace behaviors (e.g., bullying, aggression, and theft), which maintain a direct link between the perpetrator and the victim, workplace ostracism is a relatively subtle and insidious form of negative workplace behavior that severs the connection between the excluder and the excluded through such means as avoiding eye contact, employing the silent treatment, and so on (Williams, 2007a; Robinson et al., 2013). Furthermore, as one of the primary sources of work stress and interpersonal stress in contemporary society, workplace ostracism can have a variety of adverse effects on individuals, including on relationships, conduct, and other emotional symptoms, all of which impair the ability of individuals to perform their daily tasks and reduce their motivation to work (Yaakobi and Williams, 2016; Anjum et al., 2018; Sao and Wadhwani, 2018; Sarfraz et al., 2019). According to Khalid et al. (2020), knowledge hoarding, an intentional act of concealing knowledge by individuals, is a beneficial strategy for ostracized employees to cope with workplace ostracism. According to the COR theory, an ostracized employee can safeguard his or her remaining organizational resources by hoarding knowledge. In addition, workplace ostracism is also a key predictor of KSBs since it decreases communication and cooperation among organization members, inhibiting KSBs (Zhao et al., 2020). Based on the COR theory and the need-threat model, a two-wave longitudinal study developed by Bhatti et al. (2022) explains that workplace ostracism will lead to an increase in knowledge hiding (Bhatti et al., 2022). In addition, using a time-lagged research design, Zhao et al. (2016) revealed that individuals preferred knowledge hiding to KSBs when workplace ostracism was higher (Zhao et al., 2016). De Clercq et al. (2019) showed that workplace ostracism would decrease KSBs. At university, Takhsha et al. (2020) demonstrated that workplace ostracism had significant negative effects on KSBs.

According to Hobfoll (1989), there are four categories of resources, namely, objects (e.g., property and cars), conditions (e.g., occupation), personal characteristics (e.g., self-efficacy and optimism), and energies (e.g., knowledge). Personal characteristic resources are valuable resources and assets that help individuals resist various kinds of stress. Previous research proved that workplace ostracism can be a distressing experience for university teachers and can increase their pressure (Eickholt and Goodboy, 2017). In times of heightened stress, ostracized university teachers deplete significant personal characteristic resources to tackle the discomfort of stress and negative emotions and may engage in fewer KSBs to protect and avoid further depletion of other individual resources, such as energy resources (knowledge). According to the gain paradox principle (Hobfoll et al., 2018), the acquisition and replenishment of resources are more valuable to the individual when the loss of resources has been severe. This principle is analogous to 'offer fuel in snowy weather': when ostracized university teachers lose a significant amount of personal characteristic resources, the acquisition and injection of new resources may be very effective at relieving teacher stress. However, workplace ostracism “severs” the social ties of an ostracized teacher from other university teachers (Williams and Nida, 2011). Thus, ostracized university teachers feel that they are unable to participate in the communicative interaction of KSBs and are therefore unable to access critical resources, information, experiences, skills, and advice in their state of resource deprivation, which in turn exacerbates the impact of negative emotions such as anxiety and stress. As a result, ostracized university teachers need to devote more resources to handle greater pressure under the combined effects of existing resource loss and blocked access to new resources. In this state, ostracized university teachers may enter a defensive mode to protect themselves, thus significantly reducing the likelihood of them engaging in KSBs (Hobfoll et al., 2018).

**Hypothesis 1**. There is a negative relationship between workplace ostracism and KSBs.

### Job burnout

Job burnout is a state of physical and mental fatigue and exhaustion that occurs when an individual is under prolonged pressure at work; it consists of the following three dimensions: emotional exhaustion, cynicism, and reduced personal accomplishment (Maslach and Jackson, 1981). By definition, individual job burnout is closely related to work stress. Numerous studies suggested that workplace ostracism can increase personal stress and can be a significant antecedent to job burnout (Anjum et al., 2018; Jahanzeb and Fatima, 2018; Farasat et al., 2021). The COR theory also posits that the individual depletion of critical resources can generate stress (Hobfoll, 1989). Workplace ostracism can threaten the social needs of individuals, such as belongingness, control, meaningful existence, and self-esteem, which are essential resources for individuals (Williams, 2007b). Other relevant studies corroborate this finding. For example, Qian et al. (2019) confirmed that workplace ostracism can lead to a loss of job resources for individuals, hence leading to job burnout. According to Loh and Loi (2018), job stressors (e.g., workplace ostracism) can deplete the resources of an employee, resulting in job burnout. Wu et al. (2012) suggested that workplace ostracism may lead to emotional exhaustion; when the needs of emotional sharing cannot be fulfilled, emotional resources are lost, thereby leading to emotional exhaustion. Drawing on the COR theory, Chen and Li (2020) found that ostracized employees must consume mental resources, which leads to emotional exhaustion. Besides, another meta-analytic study provided evidence of the positive relationship between exposure to workplace ostracism and exhibiting of emotional exhaustion (Howard et al., 2020). Similarly, university teachers, as a group with a high incidence of job burnout, are under tremendous pressure to teach, conduct research, and do other work (Wang et al., 2020); therefore, valuable resources such as self-esteem and a sense of belonging are beneficial for university teachers to relieve work stress. However, workplace ostracism depletes these key resources, causing university teachers to experience significant psychological stress in their emotions, relationships, and self-assessment and exhibit signs of burnout such as high emotional exhaustion, cynicism, and low personal fulfillment (Tutar et al., 2021).

According to the COR theory, resource-poor individuals are more vulnerable to resource loss, and the loss of resources will spiral, with their loss spirals gaining momentum and negative impacts becoming more intense (Hobfoll et al., 2018). According to Maslach et al. (2001), job burnout excessively depletes the physical and psychological resources of individuals, mainly manifesting as physical and mental exhaustion, indifference to work, and a low sense of achievement due to their inability to compete in the job. Moreover, Zhang et al. (2016) pointed out that university teachers with high job burnout are less likely to engage in KSBs because job burnout diminishes their enthusiasm for their work and their confidence and courage to share knowledge. On the one hand, individuals experiencing resource loss find it difficult to participate in effective resource investment behavior, which makes it more difficult to prevent further resource loss. On the other hand, the principle of primacy of loss dictates that once the resources of an individual have been damaged, it triggers a response of stress and tension, especially in the case of resource loss spirals, where the individual (or organization) has fewer resources to spend on preventing resource loss (Hobfoll, 1989; Hobfoll et al., 2018). In light of this, we assume that university teachers with high job burnout are resource-poor individuals vulnerable to the negative effects of resource loss spirals when their resources are compromised. Accordingly, university teachers with high job burnout have to dedicate extensive resources to halting further resource loss, but they also have a decreasing amount of resources available to stop depletion during stressful situations. KSBs are extra-role behaviors that require additional resources, time, and effort on the part of university teachers. Therefore, under the influence of the resource loss spiral, when university teachers feel very burnt out, they lack the extra resources and energy to engage in KSBs (Ali et al., 2021).

**Hypothesis 2**. Job burnout mediates the relationship between workplace ostracism and KSBs.

### Job satisfaction

Job satisfaction is a pleasurable or positive emotional state resulting from the job or work experience of an individual (Locke, 1976). According to Chung and Kim (2017), job satisfaction mediates the relationship between workplace ostracism and innovative behavior, i.e., ostracized individuals lack communicative interaction with members of the organization and generate more negative job evaluations in a high-interpersonal-stress work environment. Workplace ostracism is a process of exclusion characterized by the rejection of interpersonal communication in the workplace, both verbally (by refusing to speak to ostracized university teachers in the workplace) and non-verbally (not making eye contact with ostracized university teachers in the workplace) (Eickholt and Goodboy, 2017). As a result, ostracized university teachers develop a sense of 'social death' (Williams, 2007a), develop negative attitudes toward others and the organization while working in a constant state of low and distressing emotions (Mao et al., 2018), and focus on the negative aspects of their work, all of which lead to lower job satisfaction (He et al., 2020).

There is a positive correlation between job satisfaction and KSBs (Sang et al., 2020). When university teachers have high job satisfaction, they tend to engage in KSBs (Bibi and Ali, 2017). According to Naz and Li (2019), job satisfaction enables people with high emotional intelligence to proactively share information and knowledge with other members of the workplace by regulating their own emotions. Based on positive reciprocal exchanges, high-emotional-quotient employees will show more positive KSBs in return for the job satisfaction they gain in the workplace. Previous research also highlighted that job satisfaction, as a source of intrinsic motivation for individuals, plays a vital role in KSBs (Kucharska et al., 2018). Under the positive impact of high job satisfaction, identification of individuals with the organization and work engagement is significantly higher, and their willingness to share knowledge increases. Therefore, we posit that job satisfaction is a significant predictor of KSBs of university teachers and that their motivation to engage in KSBs will grow as their job satisfaction increases.

**Hypothesis 3**. Job satisfaction mediates the relationship between workplace ostracism and KSBs.

### The chain mediating role of job burnout and job satisfaction

A severe consequence of job burnout is reduced job satisfaction (Ran et al., 2020). According to Herzberg et al. (1959), the main motivational factors that promote individual job satisfaction include a sense of job achievement, social identification, and job responsibility. The sense of achievement and sense of responsibility are seen as valuable resources for the individual (Russell et al., 2017; Hobfoll et al., 2018). An empirical study suggested that, of the three dimensions of job burnout, emotional exhaustion directly and negatively predicted job satisfaction, with employees experiencing lower job satisfaction when they experienced stress or depleted psychological energy; cynicism indirectly reduced job satisfaction by reducing personal accomplishment; and if employees treated customers with indifference and neglect, they may not be able to maintain positive emotions, thus reducing their sense of personal accomplishment and leading to a decline in their job satisfaction (Lee and Ok, 2012). Moreover, a recent study examined how job burnout affects job satisfaction from the perspective of COR theory, implying that continuous resource depletion or scarcity for individuals can cause job burnout, which lowers job satisfaction (Anser et al., 2020). Similarly, a study by Chang and Yi (2018) also adopts the perspective of COR theory and suggests that job burnout mediates job craft and job satisfaction; the study concludes that reducing job burnout can increase job satisfaction. Therefore, we hypothesize that job burnout of university teachers is a significant antecedent in predicting job satisfaction.

**Hypothesis 4**. Job burnout and job satisfaction sequentially mediate the association between workplace ostracism and KSBs.

**Figure 1:**
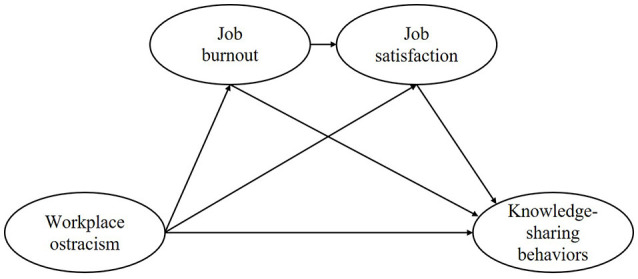
Conceptual model of the chain mediating role of job burnout and job satisfaction on the associations between workplace ostracism and knowledge-sharing behaviors.

In summary, we propose a chain mediating model (Figure 1) based on the COR theory to explain the underlying mechanisms influencing the KSBs of university teachers in the higher education sector. Specifically, this study hypothesizes that ostracized university teachers deplete a significant portion of critical resources, fueling job burnout in a context of continuous resource depletion and stress. Job burnout further depletes the emotional resources of university teachers, weakens their sense of job responsibility, reduces their sense of personal accomplishment, and lowers their job satisfaction in this negative state, ultimately reducing their engagement in KSBs.

## Method

### Participants and procedures

A total of 415 university teachers responded to the online survey between October and November 2021. Some participants were removed mainly for two reasons: first, they were double-filled and had incorrect basic information (e.g., age and teaching experience did not match, *n* = 5) and, second, they tended to answer regularly (e.g., selecting the same answer in multiple consecutive options or throughout the questionnaire, *n* = 22). Finally, 388 university teachers (155 male and 233 female) were included in the analysis. The average age of participants is 34.36 years (*SD* = 7.56), and the working span of their service is 9.60 years (*SD* = 8.35). As for the educational backgrounds of participants, university teachers with bachelor's degrees, master's degrees, and doctoral degrees were 24.2% (*n* = 94), 60.6% (*n* = 235), and 15.2% (*n* = 59), respectively. Regarding the distribution of professionals, most faculty members are full-time, with research and teaching duties. In our sample, there are 130 teaching assistants (33.5%), 150 lecturers (38.7%), 69 associate professors (17.8%), and 17 professors (4.4%). There are also faculty members who serve in both teaching and administrative positions, such as deans of colleges and directors of departments. There are 22 (5.7%) people.

Ethical approval was obtained from the ethical committee of the affiliation of the corresponding author. The participants in this study were invited to provide data through an online questionnaire. Participant gathering and data analysis were anonymous. To balance the linguistic equivalence between Chinese and English, the whole online questionnaire was translated into a Chinese version by two bilingual teachers. Then, we made some simple adjustments to language and wording after checking the equivalence of the original and the translation versions to help participants understand the content. The participants signed informed consent and completed the survey after reading the instructions.

### Measures

#### Workplace ostracism

Perceived workplace ostracism of university teachers was assessed using a 10-item workplace ostracism scale (WOS) developed by Ferris et al. (2008). This scale was validated in the Chinese context (Deng et al., 2021). The 5-point Likert scale focuses on the current self-reported degree of workplace ostracism of university teachers (from 1 = strongly disagree to 5 = strongly agree). Sample items are “You noticed others would not look at you at work” and “Others at work shut you out of the conversation.” The average score for all items was calculated, and a higher score indicates more workplace ostracism. In the present study, the Cronbach's alpha coefficient was 0.96.

#### Job burnout

Job burnout of university teachers was assessed using the Maslach Burnout Inventory-General Survey (MBI-GS; Maslach and Jackson, 1981). All participants answered the Chinese version, which was validated in the Chinese population (Li and Shi, 2003). University teachers indicated how frequently they experienced job burnout on a 5-point Likert scale (1 = never, 2 = rarely, 3 = usually, 4 = often, and 5 = frequently). This variable consists of the following three dimensions: emotional exhaustion (5 items), cynicism (4 items), and reduced personal accomplishment (6 items; R). The mean scores of three dimensions were calculated and used in the analyses, and a higher score indicates greater university teachers' job burnout. Sample items are “I feel emotionally drained from my work” and “I feel burned out from my work.” The Cronbach's alpha coefficient was 0.94 in this study.

#### Job satisfaction

Job satisfaction of university teachers was measured using the “Michigan Organizational Assessment Questionnaire-Job Satisfaction Subscale (MOAQ-JSS)” that was developed by Cammann et al. (1979). This scale was validated in the Chinese context (Qi, 2018). This scale consists of 3 items rated on a 5-point scale from 1 (strongly disagree) to 5 (strongly agree). The following are the three items: “All in I am satisfied with my job,” “In general, I don't like my job (R),” and “In general, I like working here.” The average score for the three items was calculated, and a higher score indicates more job satisfaction. The Cronbach's alpha coefficient was 0.94 for the present study.

#### Knowledge-sharing behaviors

This variable was measured using 8 items knowledge-sharing behaviors scale (KSBS) developed by Lu et al. (2006) and validated in the Chinese context (Zheng and Fu, 2018). This scale was rated on a 5-point scale from 1 (strongly disagree) to 5 (strongly agree). Sample items are “After learning new knowledge useful to work, I promote it to let more people learn it” and “So long as the other colleagues need it, I always tell whatever I know without any hoarding.” The average score for all items was calculated, and a higher score indicates more KSBs. The Cronbach's alpha coefficient was 0.90 in this study.

### Control variables in KSBs

In the study, several control variables from the literature reviews were included (Zhao et al., 2016, 2020). Gender (1 = male and 2 = female), age, and level of education (1 = bachelor's degree, 2 = master's degree, and 3 = doctoral degree) of university teachers were included as covariates in all the analyses.

### Data analysis

*SPSS* 21.0 and *Mplus 8.0* were used to test the data of the study. First, descriptive statistics and correlation analysis were performed in *SPSS 21.0* to explore the correlations between the key variables. Second, the hypothesized model was examined *via* structural equation modeling (SEM) in *Mplus 8.0* (Cheung and Lau, 2017). The values of the comparative fit index (CFI; acceptable > 0.90, good > 0.95), root mean square error of approximation (RMSEA; acceptable < 0.08, good < 0.05), and standardized root mean square residual (SRMR; acceptable < 0.08, good < 0.05) were used to judge the model fit (Steiger, 1990). The bootstrapping (N = 5,000) technique and its 95% confidence interval (CI) were employed to determine the significance of the mediation effect. When the 95% CI for an indirect effect did not include 0, the indirect effect was significant.

## Results

### Confirmatory factor analysis

Following the recommendations of Conway and Lance (2010), we conducted four CFAs to examine the discriminant validity of the four self-reported variables of university teachers. As shown in Table 1, the assumed four-factor model (M_4_, workplace ostracism, job burnout, job satisfaction, and KSBs) provided a better fit to the data than any other model, including a model in which workplace ostracism and job burnout were combined but job satisfaction and KSBs were modeled as separate factors (*M*_3_), RMSEA = 0.10, CFI = 0.85, TLI = 0.83, and SRMR = 0.17, including a model in which workplace ostracism, job burnout, and job satisfaction were combined and KSBs were modeled as separate factors (*M*_2_), RMSEA = 0.12, CFI = 0.74, TLI = 0.72, and SRMR = 0.18. The four-factor model was also superior to a one-factor model that combined all four variables into one factor (*M*_1_), RMSEA = 0.14, CFI = 0.69, TLI = 0.65, and SRMR = 0.19. Therefore, the fit results support the proposed four-factor model (*M*_4_), RMSEA = 0.05, CFI = 0.96, TLI = 0.95, and SRMR = 0.07.

**Table 1:** Model fit results from confirmatory factor analyses.

**Variable**	**χ^2^**	**df**	**Δχ^2^ (Δdf)**	**CFI**	**TLI**	**RMSEA**	**90%CI**	**SRMR**
M4: WO; JB; JS; KSB	1,188.467	560	–	0.96	0.95	0.05	[0.046, 0.055]	0.07
M3: WO + JB; JS; KSB	2,531.084	566	1,342.61 (6)	0.85	0.83	0.10	[0.091, 0.098]	0.17
M2: WO + JB + JS; KSB	3,880.821	568	2,692.35 (8)	0.74	0.72	0.12	[0.119, 0.126]	0.18
M1: WO + JB + JS + KSB	4,607.414	571	3,418.95 (11)	0.69	0.65	0.14	[0.131, 0.139]	0.19

WO, workplace ostracism; JB, job burnout; JS, job satisfaction; KSB, knowledge-sharing behaviors; CFI, comparative fit index; TLI, Tucker-Lewis index; RMSEA, root-mean-square error of approximation; SRMR, standardized root mean square residual. All Δχ^2^ are significant at *p* < 0.001.

### Descriptive statistics and correlations

Means, standard deviations, correlations of the variables are presented in Table 2. Specifically, workplace ostracism was positively associated with job burnout but negatively associated with job satisfaction and KSBs. Besides, job burnout was negatively associated with job satisfaction. All associations were in the expected direction and provided preliminary support for the hypothesized relationships.

**Table 2:** Means, standard deviations, and correlations among variables.

	**1**	**2**	**3**	**4**	**5**	**6**	**7**	**8**	**9**	**10**
**Covariates**										
1. Gender										
2. Age	−0.13^**^									
3. Level of education	−0.16^**^	0.26^***^								
**Key variable**										
4. Workplace ostracism	−0.11^*^	0.12^*^	0.02							
5. Job burnout	−0.07	−0.02	−0.02	0.34^***^						
6. Emotional exhaustion	−0.09	−0.05	0.01	0.28^***^	0.85^***^					
7. Cynicism	−0.08	−0.04	−0.03	0.35^***^	0.88^***^	0.77^***^				
8. Reduced personal accomplishment	−0.00	0.05	−0.03	0.21^***^	0.74^***^	0.33^***^	0.46^***^			
9. Job satisfaction	0.01	−0.00	−0.08	−0.27^***^	−0.40^***^	−0.27^***^	−0.35^***^	−0.36^***^		
10. Knowledge−sharing behaviors	0.08	−0.05	−0.12^*^	−0.37^***^	−0.36^***^	−0.22^***^	−0.33^***^	−0.34^***^	0.42^***^	
*M*	1.6	34	1.9	1.3	2.4	2.7	2.2	2.4	3.8	4.0
*SD*	0.5	7.6	0.6	0.5	0.7	1.0	1.0	0.8	0.7	0.5

^*^p < 0.05, ^**^p < 0.01, ^***^p < 0.001. Gender: 1 = male, 2 = female; Level of education: 1 = bachelor's degree, 2 = master's degree, 3 = doctoral degree.

### Testing the direct and mediational pathways

#### Direct effects

We first used SEM (bootstrapping with 5,000) to test the direct effect of workplace ostracism on KSBs. The model fit the data well: χ^2^ = 373.000, *df* = 179, and RMSEA = 0.053 with 90% CI = [0.045, 0.060], CFI = 0.968, TLI = 0.963, and SRMR = 0.053. Model results were consistent with Hypothesis 1, indicating that higher workplace ostracism was associated with lower levels of KSBs (β = −0.35, *SE* = 0.10, *p* < 0.001, 95% CI = [−0.543, −0.161], *B* = −0.37) after controlling for gender, age, and the education level of the university teachers. Thus, Hypothesis 1 was supported.

#### Mediational effects

**Figure 2:**
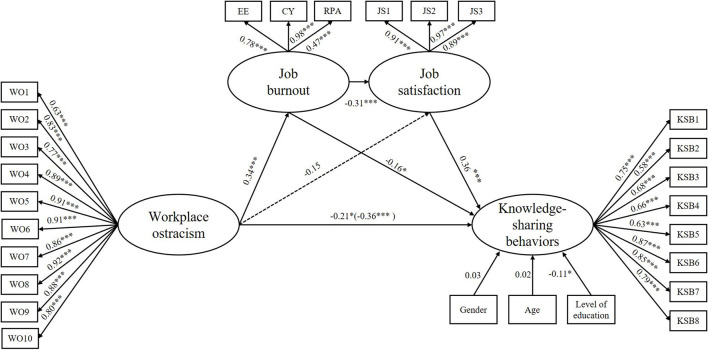
The chain mediating effect of job burnout and job satisfaction in the relationship between workplace ostracism and knowledge-sharing behaviors. Standardized coefficients are reported; *p < 0.05, **p < 0.01, ***p < 0.001. EE, emotional exhaustion; CY, cynicism; RPA, reduced personal accomplishment. Gender, age, and level of education in knowledge-sharing behaviors were included as covariates. Value in parenthesis represents the total effect.

Next, we used SEM (bootstrapping with 5,000) to examine the mediating roles of job burnout and job satisfaction. The mediation model fit the data well: χ^2^ = 673.136, *df* = 309, and RMSEA = 0.055 with 90% CI = [0.049, 0.061], CFI = 0.954, TLI = 0.948, and SRMR = 0.059. As shown in Figure 2, higher workplace ostracism was associated with low KSBs (β = −0.21, *SE* = 0.08, *p* = 0.013, 95% CI = [−0.363, −0.042], *B* = −0.21) and higher job burnout (β = 0.34, *SE* = 0.06, *p* < 0.001, 95% CI = [0.231, 0.446], *B* = 0.52), and higher job burnout was associated with lower KSBs (β = −0.16, *SE* = 0.06, *p* = 0.010, 95% CI = [−0.273, −0.037], *B* = −0.11). Furthermore, higher job satisfaction was associated with more KSBs (β = 0.36, *SE* = 0.07, *p* < 0.001, 95% CI = [0.227, 0.499], *B* = 0.27); however, workplace ostracism was not associated with job satisfaction (β = −0.15, *SE* = 0.09, *p* = 0.093, 95% CI = [−0.322, 0.031], *B* = −0.21).

Finally, as shown in Table 3, the data from the bias-corrected bootstrapping test of the mediating effect indicated that the value of the total mediating effect was significant (*B* = −0.15, *SE* = 0.04, *p* < 0.001, 95% CI = [−0.237, −0.073]). We found that job burnout mediated the link between workplace ostracism and KSBs (*B* = −0.06, *SE* = 0.02, *p* = 0.022, 95% CI = [−0.107, −0.015]), but job satisfaction did not significantly mediate the link between workplace ostracism and KSBs (*B* = −0.06, *SE* = 0.03, *p* = 0.099, 95% CI = [−0.127, 0.006]). More importantly, the chain mediating effect of job burnout and job satisfaction was significant (*B* = −0.04, *SE* = 0.01, *p* = 0.003, 95% CI = [−0.074, −0.020]). Thus, Hypotheses 2 and 4 were supported, but Hypothesis 3 was not.

**Table 3:** The specific indirect effect for each indirect pathway in the chain mediation model.

**Specific pathways**	**Bias-corrected bootstrapped**		
**tested in the chain mediation model**	**estimates for the effects**		
	**Standardized**	**SE**	**95%CI**
**Direct pathway**			
Workplace ostracism → Knowledge-sharing behaviors	–**0.21**	**0.08**	**[**–**0.363**, –**0.042]**
**Indirect pathway**			
IND1: Workplace ostracism → JB → Knowledge-sharing behaviors	–**0.06**	**0.02**	**[**–**0.107**, –**0.015]**
IND2: Workplace ostracism → JS → Knowledge-sharing behaviors	−0.06	0.03	[−0.127, 0.006]
IND3: Workplace ostracism → JB → JS → Knowledge-sharing behaviors	–**0.04**	**0.01**	**[**–**0.074**, –**0.020]**
Diff1 = IND1 – IND2	0.00	0.04	[−0.075, 0.088]
Diff2 = IND1 – IND3	−0.02	0.03	[−0.070, 0.046]
Diff3 = IND2 – IND3	−0.02	0.04	[−0.083, 0.063]
**Total indirect effect**	–**0.15**	**0.04**	**[**–**0.237**, –**0.073]**
**Total effect**	–**0.36**	**0.12**	**[**–**0.582**, –**0.141]**

Standardized coefficients are reported; the significant results are in bold. JB, job burnout; JS, job satisfaction.

## Discussion

We provide empirical evidence to support the theoretical link between workplace ostracism and KSBs. In particular, we find that job burnout mediates the relationship between workplace ostracism and KSBs. More importantly, job burnout and job satisfaction sequentially mediate the association between workplace ostracism and KSBs. These findings have several important theoretical and managerial implications.

First, this study finds that workplace ostracism negatively predicts KSBs in Chinese university teachers. The study by Zaman et al. (2021) provided support for Hypothesis 1 that workplace ostracism has a negative and significant relationship with KSBs. Previous research viewing workplace ostracism as a type of interpersonal interaction has found that ostracized employees are influenced by negative reciprocity beliefs and adopt knowledge-hiding behaviors in reaction to workplace ostracism (Zhao et al., 2016). The current study is based on COR theory and uses an individual resource gain/loss perspective to explore the motivations behind the reduction of ostracized university teachers in KSBs. It not only complements the study by Zhao et al. (2016) at a rational level and demonstrates that there are emotional and sensible distinctions among the driving mechanisms behind KSBs, but it also provides an appropriate theoretical framework to further elucidate the relationship between workplace ostracism and KSBs in higher education. To the best of our knowledge, previous studies on KSBs in higher education have mainly focused on their positive predictors, such as individual (trust, psychological empowerment, and affective commitment), organizational (organizational memory and top management support), and technological (KM system quality and HRM practices) factors (Tan, 2016; Feiz et al., 2019; Naeem et al., 2019), resulting in a study gap about the barrier of KSBs from the perspective of interpersonal mistreatments and individual resource gains/losses at work.

Second, this study proves that job burnout acts as a mediator in the linkage between workplace ostracism and KSBs, i.e., that workplace ostracism has an indirect and negative effect on KSBs *via* job burnout; thus, Hypothesis 2 is verified. Although previous research has proven that workplace ostracism can negatively affect KSBs (Takhsha et al., 2020), there is less discussion of job burnout as mediating the relationship between workplace ostracism and KSBs in higher education. Hence, our research has further extended the limited literature. Based on COR theory and using the perspective of teacher resource deprivation, this study confirms that workplace ostracism depletes university teachers of significant amounts of valuable resources, thus triggering job burnout. Al-Kurdi et al. (2020) found that academics are particularly passionate about KSBs in the context of higher education because they believe they have the resources and time to engage in such behaviors. University teachers with high job burnout are part of a resource-poor group that is more vulnerable to the adverse effects of resource loss spirals, and they no longer have the extra resources and energy to engage in KSBs amidst the situational pressures of further resource deprivation and failed resource acquisition.

Third, surprisingly, while the data indicate that job satisfaction predicts KSBs, job satisfaction failed to mediate the relationship between workplace ostracism and KSBs; that is, workplace ostracism could not indirectly influence KSBs through job satisfaction, which is inconsistent with Hypothesis 3 and contradicts earlier research (Eisenberger et al., 2003). One possible explanation is that certain variables may affect the link between workplace ostracism and job satisfaction. Although workplace ostracism is considered a resource drain, an additional study found that social networking sites can assist ostracized individuals in communicating work-related information to other organization members, as indirect communication channels can promote positive feelings toward work, alleviate the discomfort associated with direct social interactions, and encourage interpersonal interactions (Zhang and Leung, 2015). Chung and Kim (2017) further pointed out that even if ostracized university teachers cannot interact and communicate directly with others, they may mitigate the negative effects of workplace ostracism on job satisfaction through social networking activities. As a pathway for indirect communication, social networking can meet the needs of university teachers for resources, information, and social interaction, thus reducing the negative impact of workplace ostracism on their job satisfaction.

Finally, another intriguing finding of this study is that although workplace ostracism cannot directly predict job satisfaction, it can indirectly influence job satisfaction through job burnout. Previous studies have indicated that job burnout has a negative and significant effect on job satisfaction (Chong and Monroe, 2015; Lu and Gursoy, 2016; Gomez-Garcia et al., 2021). In addition, we also find that the link between workplace ostracism and KSBs is sequentially mediated by job burnout and job satisfaction, which further confirms the underlying mechanism linking workplace ostracism and KSBs. However, no single factor can explain the intrinsic mechanisms of the participation of university teachers in KSBs. Therefore, the complex interaction of multiple factors, such as interpersonal relationships, work pressure, and work attitudes in the workplace, needs to be considered to provide a more comprehensive and rational explanation for the intrinsic motivation of university teachers to engage in KSBs.

## Practical implications

This study revealed the potential mechanism for workplace ostracism and decreased KSBs and has practical implications for university management. First, lowering workplace ostracism inside institutions will help enhance the KSBs of university teachers. In organizations, workplace ostracism has become a “severely common issue.” Knowledge-intensive organizations such as universities can gain core competitive force results from KSBs. Thus, to decrease workplace ostracism in universities, administrators should make policies and procedures that prevent university teachers from being ostracized (Jiang et al., 2021). For example, they can take actions such as building friendly and close teacher groups, including redesigning procedures and introducing highly interdependent tasks. Furthermore, ostracized university teachers could be made comfortable by discussing workplace ostracism with their deans, which would allow deans to appropriately address workplace ostracism and help ostracized university teachers to better respond to it. More importantly, the university should prioritize the development of an inclusive and nondiscriminatory culture (Fatima et al., 2019) and constantly strive for an environment of solidarity, collegiality, and team cohesion (Tan, 2016) to minimize the occurrence of workplace ostracism in the university.

Second, this research indicates a chain mediating effect of job burnout and job satisfaction between workplace ostracism and KSBs. This pattern suggests that maintaining a low level of job burnout and a high level of job satisfaction might also promote KSBs of university teachers. Thus, universities need to focus on job burnout and job satisfaction. Since workplace ostracism depletes the resources of university teachers and causes job burnout, it is crucial to provide the resources needed by exhausted university teachers. For example, deans and colleagues can give ostracized university teachers more mental support and replenish their emotional resources (Chen and Li, 2020). In addition, universities should establish service organizations to give assistance and psychological guidance to ostracized university teachers, alleviate their negative emotions, and provide a platform to solve their problems.

## Limitations and future research

There are two limitations to our current research. First, the data for our study were cross-sectional and did not reveal a causal relationship between workplace ostracism and KSBs. Thus, future studies could adopt a longitudinal design to explore the underlying mechanisms of workplace ostracism and KSBs in depth. Second, this study mainly examined the relationship between workplace ostracism and KSBs based on an individual-level perspective, verifying the chain mediating role of job burnout and job satisfaction. However, KSBs of university teachers are multifactorial and complex processes; thus, future research could further explore the relationship between workplace ostracism and KSBs from different perspectives, such as the organizational and leadership levels.

## Conclusion

Based on the conservation of resources theory, we examined the potential mechanism underlying the link between workplace ostracism and KSBs. After controlling for KSBs, workplace ostracism significantly and negatively predicts KSBs. In addition, workplace ostracism indirectly influences KSBs by sequentially increasing job burnout and undermining job satisfaction. More importantly, this finding extends our understanding of the mechanisms underlying workplace ostracism that influence KSBs in higher education.

## Data availability statement

The raw data supporting the conclusions of this article will be made available by the authors, without undue reservation.

## Ethics statement

Ethical approval was obtained from the Ethical Committee of corresponding author's affiliation (Guangzhou University). The patients/participants provided their written informed consent to participate in this study. Written informed consent was obtained from the individual(s) for the publication of any potentially identifiable images or data included in this article.

## Author contributions

G-HW and HL: conceptualization, investigation, and resources. G-HW and J-HL: data curation, formal analysis, methodology, software, and writing—original draft. HL: project administration. CZ and HL: supervision, writing—review, and editing. All authors contributed to the article and approved the submitted version.
