# Corrigendum: The association between workplace ostracism and knowledge-sharing behaviors among Chinese university teachers: The chain mediating model of job burnout and job satisfaction

**DOI:** 10.3389/fpsyg.2023.1156498

**Published:** 2023-02-23

**Authors:** Guang-Hui Wang, Jia-Hui Li, Hui Liu, Cristina Zaggia

**Affiliations:** ^1^School of Education (Teachers College), Guangzhou University, Guangzhou, China; ^2^Department of FISPPA, University of Padova, Padova, Italy

**Keywords:** workplace ostracism, job burnout, job satisfaction, knowledge-sharing behaviors, conservation of resources theory

In the published article, there was an error in Literature review and hypotheses, Relationship between workplace ostracism and KSBs, Hypothesis 1. This sentence previously stated, “There is a positive relationship between workplace ostracism and KSBs”. The corrected sentence appears below:

**Hypothesis 1**. There is a negative relationship between workplace ostracism and KSBs.

The authors apologize for this error and state that this does not change the scientific conclusions of the article in any way. The original article has been updated.

